# Antidepressant use at the threshold: people prescribed antidepressants around the time of a dementia diagnosis

**DOI:** 10.1093/ageing/afag221

**Published:** 2026-07-27

**Authors:** Mohamed Heybe, Shreya Verma, Beatriz Pozuelo Moyano, Rob Stewart, Christoph Mueller, Katrina A S Davis

**Affiliations:** Psychological Medicine, King’s College London Institute of Psychiatry Psychology & Neuroscience, London, United Kingdom; Academic Psychiatry, King's College London Institute of Psychiatry Psychology & Neuroscience, London, United Kingdom; Service of Old Age Psychiatry, Department of Psychiatry, Lausanne University Hospital, Prilly, VD, Switzerland; Department of Psychiatry, University of Lausanne, Lausanne, VD, Switzerland; Psychological Medicine, King’s College London Institute of Psychiatry Psychology & Neuroscience, London, United Kingdom; NIHR Biomedical Research Centre: Maudsley, South London and Maudsley NHS Foundation Trust, London, United Kingdom; NIHR Biomedical Research Centre: Maudsley, South London and Maudsley NHS Foundation Trust, London, United Kingdom; Department of Old Age Psychiatry, King's College London Institute of Psychiatry Psychology & Neuroscience, London, United Kingdom; Psychological Medicine, King’s College London Institute of Psychiatry Psychology & Neuroscience, London, United Kingdom; NIHR Biomedical Research Centre: Maudsley, South London and Maudsley NHS Foundation Trust, London, United Kingdom

**Keywords:** dementia, prescribing, depression, electronic health records, natural language processing, older people

## Abstract

**Background:**

Antidepressant use is common in people with dementia, and many start around the time of diagnosis. We hypothesised that those who commenced antidepressants at dementia diagnosis started for dementia-related symptoms, distinct from those with longstanding prescriptions, resulting in different characteristics in electronic records.

**Methods:**

We carried out a retrospective cohort study using linked primary care (Lambeth DataNet) and mental health (Clinical Record Interactive Search) data for patients with dementia in south London, UK in 2009–22. Antidepressant prescription starting within one year before or after dementia diagnosis identified as ‘new’. Dementia and anxiety were described using coded fields, a rating scale for neuropsychiatric symptoms and natural language processing of full-text.

**Results:**

Of 3713 patients with dementia, 28% were prescribed an antidepressant in the year of dementia diagnosis. 42% of these were ‘new’ prescriptions. Compared to the no antidepressant group, ‘new’ starters were more likely to be female (odds ratio (OR) 1.33, 95% 1.08–1.64) and have vascular dementia (OR 1.44, 1.13–1.83). Compared to longstanding antidepressant group, ‘new’ had similar depression and anxiety symptoms, but fewer coded mentions (OR 0.50, 0.39–0.65), especially those ‘new’ from non-White ethnicities. Deprescribing was equally unlikely in ‘new’ and longstanding (6.3% vs 5.5% per year of follow-up).

**Conclusion:**

There is a high prevalence and lack of deprescribing, despite guidelines cautioning antidepressants. This points to unmet needs at the threshold of dementia. Clinicians should ensure good documentation of indication for antidepressants and proactive medication reviews for people with dementia.

## Graphical Abstract

**Graphical Abstract:**
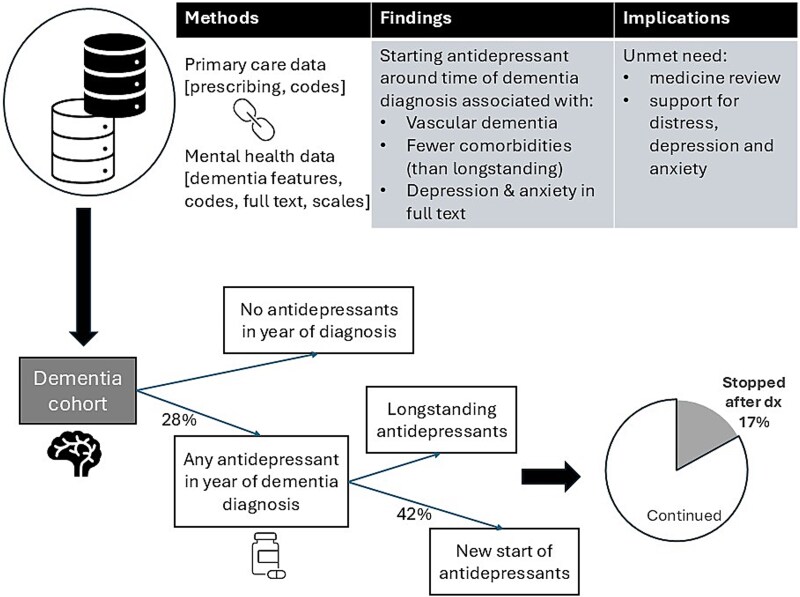

## Key Points

- Antidepressants have poor evidence of efficacy, and the possibility of adverse effects, in dementia.
- In the year of a dementia diagnosis 28% had an antidepressant prescribed, nearly half (42%) started less than a year before.
- Natural language processing showed depression symptoms in those taking antidepressants high, regardless of when commenced.
- Deprescription of antidepressant was low, at 6% per year of follow-up.

## Background

The threshold period where dementia is suspected, investigated and newly confirmed can be a confusing and distressing time. The differential diagnosis for symptoms of depression and anxiety during this time includes depressive and anxiety disorders, neuropsychiatric symptoms of dementia and reactions to the uncertainty during the assessment process and changing social roles [1, 2]. Depression is a risk factor for dementia, and symptoms of depression often feature as a prodrome of dementia [3, 4]. Depression may also have distinct neurobiological features when appearing in someone with dementia [5, 6].

The prevalence of antidepressants in adults of all ages is ~23%, rising with female sex and older age [7, 8]. Some people experiencing depression and anxiety around the time of dementia diagnosis may benefit from antidepressants [9]; however, prescribing for older people, particularly those with dementia, has elevated risks [10]. Non-antidepressant strategies are considered first-line in people with dementia, including psychological treatment for depression and anxiety, personalised activities for agitation and personalised sleep management for insomnia [1].

We aimed to characterise antidepressant prescribing around the time of dementia diagnosis, the extent to which depression and anxiety were documented, and the profiles of individuals who had recently initiated an antidepressant, especially those without a recorded diagnosis of depression or anxiety. Our findings aim to support guidance and resource planning for the clinical challenge of managing mental health symptoms in people when dementia is suspected or recently diagnosed.

## Methods

We assembled a retrospective cohort of people with dementia and identified people prescribed antidepressants in the years around diagnosis. Documentation of depression or anxiety was studied, alongside demographics, dementia features and cessation of antidepressant.

### Data source

We used electronic health record data from the Clinical Record Interactive Search (CRIS) at South London and Maudsley NHS Foundation Trust (SLaM) [11], and a pre-established linkage to Lambeth DataNet (LDN). CRIS provides pseudonymised structured and free text data from SLaM, a provider of dementia and mental health care for four London boroughs [12]. LDN provides structured data about people registered with a GP in the socially and ethnically diverse London Borough of Lambeth (about 405 000 residents), one of SLaM’s catchment boroughs [13]. Natural Language Processing (NLP) has been deployed extensively in CRIS to extract a range of entities from text. Linkage includes all eligible Lambeth patients unless they have opted out of CRIS or LDN. Linkage and extraction was by the CRIS Linkage Service; investigators also had access to CRIS.

### Sample

We selected patients with a documented dementia diagnosis in LDN or CRIS as described previously [11] using the ICD-10 fields in CRIS and Read codes in LDN. The date of the first documented diagnosis of dementia was the index date, and eligible patients had an index date between 2009 and 2022, aged 65 or over, registered with a Lambeth GP and with a SLaM contact at some point from 2 years prior to 4 years post-index.

### Exposure

Antidepressant prescription was ascertained from the LDN prescription fields. The antidepressant positive group are those who were issued a prescription for antidepressants between the index date and 12 months later (the index year). We allocated each patient to one of three groups:

(i) ‘New start’: prescribed antidepressant in the index year, but not in the year 12–24 months before the index date, targeting those starting in the two years between 12 months before and 12 months after the first documented dementia diagnosis.
(ii) ‘Longstanding’: prescribed antidepressant in the index year and in the year 12–24 months before index (started >1 year before diagnosis).
(iii) ‘None’: not prescribed antidepressants in the index year.

A visual explanation of the group allocations is shown in Supplementary Appendix 1.

We identified antidepressants through the British National Formulary [14], grouped into SSRI, tricyclic (TCA) and ‘other’ antidepressants, and matched these to the codes used by LDN (drugs + medical devices, DMD, shown in Supplementary Appendix 2) to search. Using a window of observation from 2 years before diagnosis until 4 years post-diagnosis, we also noted the class of antidepressants, including the use of more than one of these in any year. Deprescription was defined as no antidepressant prescription for a complete year during follow-up. Follow-up ended after 4 years, at death, or if a patient left LDN according to registration data.

### Other variables

The derivation of all variables is shown in Supplementary Appendix 3A and timing in Supplementary Appendix 3B. Demographic factors were age at diagnosis, sex, marital status, ethnicity and neighbourhood deprivation. The Charlson comorbidity index [15] (excluding dementia) from primary care codes was a measure of physical health detailed previously [11]. Dementia factors were subtype, severity at diagnosis and difficulty of activities of daily living (from closest HoNOS 65+) [16].

### Depression, anxiety and neuropsychiatric documentation

To study diagnoses and symptoms, we used:

(i) Codes related to depression and anxiety: Coded mentions were primary care Read code in LDN from a list [17] or ICD-10 codes from the categories F32, F33 and F4 in CRIS at any date.
(ii) Full text mentions of symptoms: Using pre-existing NLP tools that identify symptoms of depression and anxiety synonyms [18], we assessed mentions in the CRIS text recorded in the 6 months either side of the index date. These are reported as being out of 21 included concepts for depression and 20 trigger words for anxiety, shown in Supplementary Appendix 4.
(iii) A rating scale: Neuropsychiatric symptoms were derived from the clinician-completed Health of the Nation Outcome Scale (HoNOS) 65+ [16] in CRIS, the closest to the index date. Sum of scores (0–4) for each of: agitation, self-injury, substance use, depression, hallucinations/delusions and other problem (which may be anxiety) up to 24, the actual distribution being a range of 0–18 with mean of 3.

### Analyses

We carried out the following analyses:

- Antidepressant factors: We identified prescription prevalence in the index year, approximate onset, grouping the cohort into ‘new start’, ‘longstanding’ and no antidepressant. We described the classes prescribed.
- Demographic and clinical factors: Characteristics were summarised using proportions or means and standard deviations for each group. Univariate regression models were used to quantify associations between groups using linear or logistic models, as appropriate for each dependent variable, giving mean difference or odds ratio with 95% confidence intervals.
- Depression/anxiety measures: These were summarised and compared between groups, as for demographic and clinical factors. Those with ‘new start’ of antidepressant were divided into two groups depending on whether they had a code for depression/anxiety, and clinical and depression/anxiety measures compared as previously.
- De-prescription: Deprescription was identified as above and in Appendix 1 in supplementary data. The number of deprescriptions per year of follow-up was calculated and compared between ‘new start’ and ‘longstanding’ groups using chi-squared tests.

Data were organised using R/R-studio and analysed in STATA and excel.

The number of demographic, clinical and anxiety/depression factors was 23 for each of the two contrasts (new vs none and new vs longstanding). To avoid false discovery due to multiple comparisons, we defined significance using the Benjamini–Hochberg corrected *P*-value [19] less than .05. Supplementary Appendix 5 shows the results with raw and corrected *P*-values. All other tables show symbols indicating the significance of the *corrected P*-values.

Post-hoc power calculations were performed, and provided in Supplementary Appendix 6.

### Missing data and bias

Items with missing data were demographic factors of marital group and ethnicity, where an unknown group was added. In clinical features, dementia subtype contained an ‘unknown’ category for ‘unspecified’ subtype and missing. Dementia severity could only be calculated if there was an MMSE or HoNOS in the 6 months either side of the index date (79%), and if not, severity was unknown. For the other HoNOS-related outcomes, we used the closest to diagnosis, which was within six months of diagnosis for 76%. When calculating neuropsychiatric scores, we excluded those with no HoNOS ratings (9%).

Those people with no records within 6 months either side of the index date would score zero on depression and anxiety NLP scores if included. We were not directly able to ascertain who these were, so to reduce the risk of bias, if a person had no MMSE nor HoNOS in that time-period (21%), they were be excluded from the NLP results. These exclusions are evident in the cohort size of Tables 2 and 3.

A completed RECORD reporting checklist [20] has been included as Supplementary Appendix 7.

## Results

### Cohort

**Figure 1:**
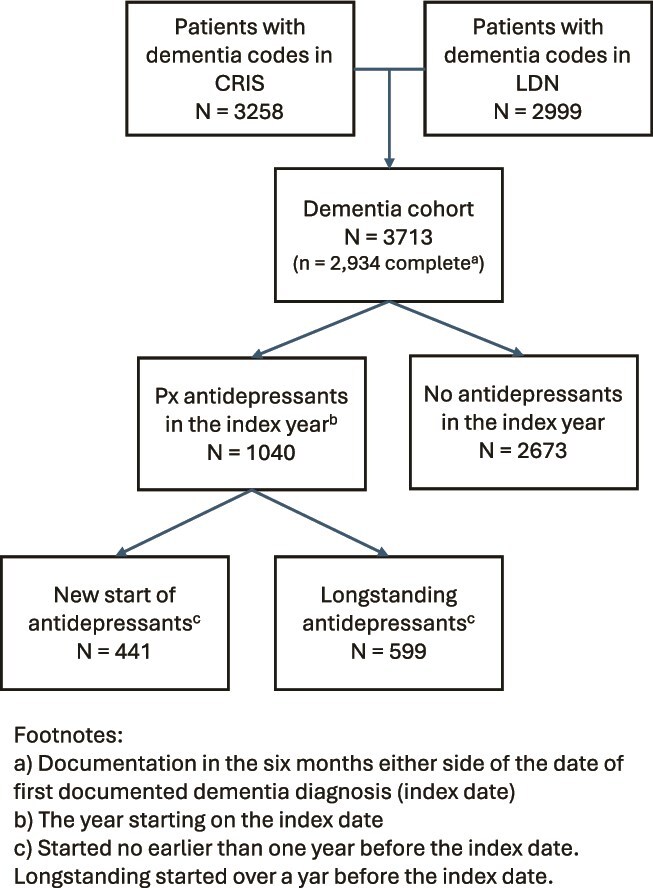
Cohort flow chart showing the formation of a dementia cohort through combining patients with a code for dementia from two sources (Clinical Records Interaction Search and Lambeth DataNet).

The cohort assembly is shown in Figure 1, with 3713 patients included in the analyses. Of the cohort, 2539 (68%) had codes for dementia in both CRIS and LDN, while 719 (19%) had codes in CRIS only, so that 87% were definitely known to have dementia in the specialist service. Documentation of at least one MMSE or HoNOS rating within 6 months either side of the index date was present in 2934 (79%). The mean follow-up time was 2.8 years (SD 2.6), with 21% alive and in LDN after four years.

Characteristics of the full cohort are in Table 1. The mean age at dementia diagnosis was 82 years, 59% were female, and the most commonly recorded ethnicity was White (61%) with 6% unknown. The most common dementia subtype was Alzheimer’s disease (62%), while 31% had mild dementia at diagnosis.

**Table 1:** Cohort characteristics, with comparison between groups defined by antidepressant status.

		Antidepressant status			New vs none		New vs Long	
Characteristics	Full cohort	New start	Long-standing	None	OR or MD (95% CI)	sig^a^	OR or MD (95% CI)	sig^a^
*N*	**3713**	441	599	2673				
Socio-demographic status								
Age at diagnosis, mean (SD)	**81.6** **(7.3)**	81.3 (7.3)	80.7 (7.6)	81.9 (7.1)	MD: −0.59 (−1.32 to 0.14)		MD: 0.67 (0.22–1.56)	
Female sex	**58.6%**	62.6%	68.8%	55.6%	**1.33 (1.08–1.64)**	^*^	0.76 (0.59–1.54)	.
Married	**30.6%**	31.3%	27.7%	30.7%	0.97 (0.77–1.23)		1.16 (0.64–1.54)	
Non-White Ethnicity	**38.7%**	37.2%	30.4%	40.8%	1.13 (0.92–1.38)		0.68 (0.53–0.88)	
More deprived^b^	**49.0%**	45.4%	48.8%	49.7%	1.12 (0.88–1.43)		1.17 (0.88–1.59)	
Health status								
Comorbidity^c^ (mean, SD)	**2.1** **(1.9)**	2.0 (1.9)	2.5 (2.1)	2.1 (1.9)	MD: −0.09 (−0.29 to 0.10)		MD **−0.51 (−0.74 to −0.27)**	^**^
Dementia factors—scores from assessment closest to diagnosis								
*Subtype*								
Alzheimer’s disease	**61.8%**	54.7%	53.9%	64.8%	**0.66 (0.53–0.80)**	^**^	1.03 (0.76–1.24)	
Vascular or mixed	**18.5%**	23.4%	19.5%	17.5%	**1.44 (1.13–1.83)**	^*^	1.26 (0.93–1.69)	
Other	**4.9%**	5.7%	7.9%	4.0%	1.43 (0.91–2.23)		0.71 (0.43–1.17)	
Unspecified	**14.8%**	16.3%	18.7%	13.7%	1.23 (0.93–1.62)		0.85 (0.61–1.17)	
*Dementia severity at diagnosis*								
Mild	**31.4%**	28.8%	30.6%	31.4%	0.92 (0.74–1.15)		0.88 (0.68–1.16)	
Moderate	**23.4%**	24.7%	23.0%	23.4%	1.10 (0.87–1.39)		1.08 (0.81–1.43)	
Severe	**24.7%**	29.3%	27.0%	24.7%	1.15 (0.89–1.50)		1.13 (0.92–1.38)	
Unknown	**20.5%**	17.2%	19.4%	20.5%	0.87 (0.66–1.13)		0.81 (0.59–1.11)	
*Functioning (n = 3387)*								
Problems with ADLs^d^	**62.0%**	66.7%	66.0%	60.3%	1.32 (1.05–1.64)	**#**	1.03 (0.79–1.35)	

^a^Significance according to corrected *P*-value: ^#^*P* < .1, ^*^P < .05 and ^**^P < .01.

^b^Index of multiple deprivations in 30% most deprived in England, based on approximate address.

^c^Based on Charlson comorbidity index (with dementia not scored).

^d^Activities of Daily Living, using ADL subscale on Health of the Nation Outcome Scale (HoNOS) 65+. Bold OR or MD signifies significant association.

### Antidepressant-related features

In the index year, there were 31 746 prescriptions for 1040 patients (28%). For those prescribed an antidepressant in the index year, 441 (441/1040, 42%) were ‘new start’, and 599 were ‘longstanding’. Both antidepressant groups had a mean follow-up of 2.9 years (SD 2.6) post index diagnosis.

Appendix 7 in supplementary data shows prescribing in the index year by antidepressant class. From the 1040, 53% received at least one prescription for an SSRI, 37% for ‘other’ antidepressants and 21% for a TCA. TCAs were prescribed for 28% (168/599) of the longstanding group and 13% (55/440) of the new start group, giving an odds ratio of 0.37 (95% confidence interval 0.26–0.51). Twelve percent received a prescription for more than one of those classes in the index year, rising to 36% when including 2 years before to 4 years after diagnosis.

### Demographic and clinical factors

Table 1 describes the three antidepressant groups. When compared with no antidepressant, the ‘new start’ group had more female patients (OR 1.33, 1.08–1.64), vascular subtype (OR 1.44, 1.13–1.83) and ADL impairment (OR 1.32, 1.05–1.64) but no relationship with age, marital status, ethnicity, deprivation or comorbidity. When compared with longstanding antidepressant, the ‘new start’ group showed a mean comorbidity score about half a point lower (−0.51, −0.74 to −0.27) than the longstanding group, and a suggestion of difference in ethnicity that did not reach significance.

### Depression/anxiety factors

Of the full cohort, 42% had at least one depression or anxiety code in GP records, and 53% had at least one recorded neuropsychiatric problem on the HoNOS, mean 3.0 on the combined scale. A mean of 1.9 symptoms of depression, and 2.0 anxiety-related words were identified by NLP. In Table 2, the ‘new start’ group had significantly higher scores on all of these measures compared to the no antidepressant group. Comparisons between ‘new start’ and ‘longstanding’ groups showed ‘new start’ had a lower prevalence of depression/anxiety codes (OR 0.48, 0.37–0.64) but slightly higher depression NLP score (2.8 vs 2.4), and no significant difference on neuropsychiatric rating or anxiety NLP scores.

**Table 2:** Depression, anxiety and neuropsychiatric factors divided by antidepressant status.

		Antidepressant status			New vs none		New vs long	
Characteristics	Full cohort	New start	Long-standing	None	Odds ratio or mean difference (95% CI)	sig^a^	Odds ratio or mean difference (95% CI)	sig^a^
No. of patients	**3713**	441	599	2673				
Depression/anxiety codes^b^ *ever* (*n* = 3713)								
Depression	**32.5%**	52.2%	68.5%	21.1%	**4.07 (3.30–5.01)**	^**^	**0.50 (0.39–0.65)**	^**^
Anxiety	**21.7%**	29.7%	44.9%	15.2%	**2.36 (1.87–2.97)**	^**^	**0.52 (0.40–0.67)**	^**^
Either	**41.9%**	64.2%	78.8%	30.0%	**4.18 (3.39–5.17)**	^**^	**0.48 (0.37–0.64)**	^**^
Neuropsychiatric rating^c^ *closest to diagnosis* (*n* = 3387)								
Combined scale mean (SD)	**3.0 (2.5)**	3.8 (2.5)	3.6 (2.7)	2.7 (2.4)	MD: **1.13 (0.87–1.40)**	^**^	MD: 0.29 (0.04–0.61)	
Natural Language Processing tool score^d^ *six months either side of diagnosis* (*n* = 2934)								
Depressive symptoms, mean (SD)	**1.9 (2.0)**	2.8 (2.3)	2.4 (2.5)	1.7 (1.7)	MD: **1.10 (0.86–1.34)**	^**^	MD**: 0.37 (0.09–0.71)**	^**^
Anxiety symptoms, mean (SD)	**2.0 (2.0)**	2.7 (2.3)	2.5 (2.4)	1.8 (1.8)	MD: **0.87 (0.66–1.14)**	^**^	MD: 0.17 (−0.10 to 0.50)	

Comparison between groups with odds ratio (OR) or mean difference (MD) with 95% confidence interval and significance based on *P*-values corrected for multiple comparisons. Bold text for OR or MD represents significant associations.

^a^Significance according to *P*-values corrected for multiple testing where ^#^*P* < .1, ^*^P < .05 and ^**^P < .01.

^b^Read codes from LDN and ICD-10 codes from CRIS.

^c^Sum of six symptom subscales from HoNOS.

^d^Using existing natural language processing tools: number of distinct depression symptoms (maximum 20); number of distinct anxiety synonyms (maximum 21).

In the ‘new starters’ group, 36% did not have depression and anxiety codes in primary or specialist databases, and may have had an alternative indication. Table 3 separates those with and without formal depression or anxiety documentation. Those from a white ethnicity and with mild dementia are more likely to have codes. Those without codes had a lower mean score for NLP depression and anxiety, but a similar neuropsychiatric rating.

**Table 3:** Characteristics of antidepressant new starters with separation by the documentation status of depression and anxiety.

	New starters	Depression or anxiety code documented in LDN or CRIS^a^		Any vs no code	
Characteristics		Any codes	No codes	Odds ratio or mean difference (95% CI)	sig^b^
*N*	**441**	283	158		
**Socio-demographic status**					
Age at diagnosis, mean (SD)	**81.3 (7.3)**	81.1 (7.2)	81.8 (7.6)	MD: −0.69 (−2.11–0.74)	
Female gender	**62.6%**	63.3%	61.0%	1.08 (0.72–1.62)	
Married	**30.7%**	29.6%	32.6%	0.87 (0.55–1.36)	
Non-White Ethnicity	**37.2%**	31.6%	47.3%	**0.51 (0.34–0.78)**	^**^
More deprived^c^	**45.4%**	46.3%	43.7%	1.11 (0.75–1.64)	
**Health status**					
Comorbidity score^d^ mean (SD)	**2.0 (1.9)**	2.0 (1.9)	2.0 (1.8)	MD: −0.04 (−0.41–0.33)	
**Dementia factors—scores from assessment closest to diagnosis**					
Subtype					
Alzheimer’s disease	**54.7%**	55.8%	52.5%	1.14 (0.77–1.69)	
Vascular or mixed	**23.4%**	22.3%	25.3%	0.84 (0.54–1.33)	
Other	**5.7%**	5.7%	5.7%	0.99 (0.43–2.30)	
Unspecified	**16.3%**	16.2%	16.5%	0.99 (0.58–1.67)	
Dementia severity at diagnosis					
Mild dementia	**28.8%**	33.2%	20.9%	**1.88 (1.19–2.97)**	^**^
Functioning (*n* = 3387)					
Problems with activities of daily living^e^	**66.7%**	65.1%	69.4%	0.82 (0.53–1.27)	
**Depression and anxiety factors**					
Neuropsychiatric symptoms^e^ *closest to diagnosis*(*n* = 3387)					
Combined scale, mean (SD)	**3.8 (2.5)**	3.9 (2.4)	3.7 (2.8)	MD: 0.18 (−0.35–0.71)	
Natural Language Processing tool score^d^ *six months either side of diagnosis* (*n* = 2934)					
Depressive symptoms, mean (SD)	**2.4 (2.3)**	2.6 (2.4)	1.9 (2.2)	**MD: 0.66 (0.22–1.11)**	^**^
Anxiety symptoms, mean (SD)	**2.2 (2.3)**	2.5 (2.4)	1.7 (2.0)	**MD: 0.79 (0.35–1.23)**	^**^

Comparison by odds ratio (OR) or mean difference (MD) with significance from pairwise regression between the documentation groups. Bold text for OR or MD signifies significant association.

^a^Read code from LDN or ICD-10 code from CRIS.

^b^Significance where ^*^P < .05 and ^**^P < .01 (no correction for multiple testing).

^c^Index of Multiple Deprivations in 30% most deprived in England, based on approximate address.

^d^Based on Charlson comorbidity index (with dementia not scored).

^e^Using scale on Health of the Nation Outcome Scale (HoNOS) 65+ scale for ADLs.

^f^Sum of symptom subscales from HoNOS.

^g^Using existing natural language processing tools: number of distinct depression symptoms (maximum 21); number of distinct anxiety synonyms (maximum 22).

### De-prescription

Of the 1040 prescribed antidepressants in the index year, 175 (17%) stopped all antidepressants for a year during follow-up. In the ‘new start’ group, 81 people (of 441) stopped antidepressants during a mean 2.9 years of follow-up: a mean of 6.3% per year of follow-up. In the ‘longstanding’ group 95 people (of 599) stopped antidepressants during a mean follow-up of 2.9 years: a mean of 5.5% per year. There was no significant difference between the groups (yearly data, *P* = .557).

## Discussion

We defined a cohort of 3713 people with dementia using routinely collected information, and defined a ‘threshold’ of dementia as one year prior to the first documented diagnosis of dementia to one year after. This is a time of suspicion and assessment, confirmation of dementia and adjustment. The prevalence of antidepressant prescription was 28% in the year of dementia diagnosis. The 441 patients who had a ‘new’ antidepressant prescription in this period accounted for 42% of those prescribed antidepressants. Compared to those without antidepressants, the ‘new’ group were more likely to be women (OR 1.33, 1.08–1.64) and to have vascular dementia (OR 1.44, 1.13–1.83). To address confounding by the risk factors of depression, we also compared with longstanding antidepressant group, showing similar in demographics except lower comorbidity in the ‘new’ group (mean 2.0 vs 2.5). Symptom measures were higher in the ‘new’ group, reaching significance for depression (2.8 vs 2.4), but not anxiety (2.7 vs 2.5). The 36% of ‘new’ group without coded entries for depression/anxiety differed in ethnicity, with those from non-White ethnicities substantially less likely to have a code (OR 0.51, 0.34–0.78), with lower NLP symptom scores (depression mean 1.9 vs 2.6, anxiety mean 1.7 vs 2.5) but similar neuropsychiatric rating (mean 3.9 vs 3.7). During follow-up of those with antidepressant prescriptions in the index year, 36% were prescribed more than one class of antidepressants and 17% (a mean of 6% per year) stopped antidepressants.

Around 30% of were prescribed TCAs, despite being cautioned in older people due to risk of cognitive decline, falls and cardiovascular issues [21, 22]. The lower use of TCAs in the ‘new’ group may mean prescribers were less keen to start TCAs in older people but may continue existing prescriptions. SSRIs were used more commonly, and though safer than TCAs, may cause QT prolongation, hyponatraemia and gastrointestinal complications in older people [23].

Vascular dementia was more common in the new start group (23%) compared to non-antidepressant group (18%), which supports the hypothesis that late-life depression may be linked to vascular pathology [24]. If depression is a marker of cerebrovascular disease, antidepressants, for which most of the evidence comes from younger adults with no vascular pathology, may be ineffective or inappropriate [5].

Depression/anxiety codes were found for 30% with no antidepressant, 79% with longstanding and 64% with ‘new’ antidepressant prescription. Therefore 36% of the ‘new’ group were without codes, similar to the 33% with no codes for depression in a UK cohort prescribed antidepressants [7]. Under-recording, a phenomenon common to dementia and mental disorders [11], will account for some of this gap. There are also alternative indications, such as pain, migraine and irritable bowel; and this may explain the higher comorbidity score in the longstanding antidepressant group. In the ‘new’ antidepressants group, some without depression/anxiety may be prescribed for distressing symptoms of dementia and pre-dementia, such as agitation, sleep disturbances or apathy [9, 25, 26], which may account for the slightly higher neuropsychiatric score despite lower NLP symptom scores in these patients.

The 36% of the ‘new’ starters with no depression or anxiety codes were significantly more likely to have a non-White ethnicity. Although care needs to be taken with this result, as it is not corrected for confounding, it may represent bias in healthcare. Previous work in the UK found that patients from Black ethnicities with depression were less likely to have this recorded and more likely to receive pharmacological treatment instead of psychotherapy [27]. In dementia care, people from minoritised communities have been prescribed more potentially harmful anticholinergic medication and antipsychotics on average, and fewer cognitive enhancers [28].

Where antidepressants were prescribed in the index year, they were continued in every year of follow-up in 83%. Give the prior evidence of poor efficacy in people with dementia [5, 29, 30], seen in the 36% who combined or switched antidepressant classes (a proxy for hard-to-treat depression [31]), and with no proven action of antidepressants against deterioration in dementia [10, 21], the lack of deprescribing suggests either a lack of medication reviews, a concern about stopping antidepressants or both [32–34]. This leads to prolonged and potentially unnecessary antidepressant use.

### Strengths and limitations

The linked data sources used in this study provide a near-unique resource for these analyses in the level of detail available from multiple care providers, including comprehensive prescription data, primary care presentation coding, specialist care scales and metadata on mental health and dementia derived from NLP. This allows comparisons that would not be possible using only coded data or only one health provider. However, the data are drawn from a single area of London, so wider generalisability cannot be assumed. Although the setting is restricted, we suggest that the themes that come up in the discussion will be similar throughout the UK and beyond.

Routinely collected data inevitably reflects recording biases, which can introduce misclassification in cohort selection and in the ascertainment of clinical characteristics. For example, we previously showed that dementia was more frequently documented when cognitive enhancers were prescribed [11], and a similar pattern may occur for depression and antidepressants, potentially inflating recorded rates of depression among those receiving such prescriptions. Our inclusion criteria may also introduce bias: requiring contact with specialist services could disproportionately select individuals with comorbid mental health difficulties, and NLP outputs depend partly on the volume of documentation, which may be longer for reasons unrelated to symptom severity. The depression NLP tool has been extensively validated in CRIS, whereas the anxiety tool is less developed and may be less reliable. In addition, using diagnosis codes as the index date with a two-year look-back may miss earlier dementia-related treatment, given the average 3.5-year interval between symptom onset and diagnosis [35], may miss the real threshold of dementia-related treatment. Limited documentation of non-pharmacological and social interventions further restricts our ability to account for these factors.

As our aim was to describe prescribing behaviour rather than infer causality, we did not adjust for confounding, and our methods did not allow for a consistent ordering of antidepressant start and symptom measures, so efficacy cannot be inferred. Due to a lack of access in LDN to source text fields in the primary care record, we cannot search for more information on decisions about prescribing, and such question may be answered best by different approaches, such as qualitative research, which could also be helpful to capture the voices of patients and carers.

### Implications for clinical practice

Our study confirmed that long-term antidepressant treatment in people with dementia has become common and that some of those starting an antidepressant around the time of diagnosis may have different reasons for starting to those with longstanding prescriptions. Clinicians should be aware, when considering an antidepressant for potentially time-limited symptoms, that it is likely to be continued, and assess whether other support available could delay commencement. Minoritised communities, such as people with non-White ethnicities in this study, appear to have less optimal treatment, so access to alternative treatment and support should be tailored to better meet these needs. Documentation of the reasons for starting an antidepressant, and sharing of this, may be necessary for ensuring all and future prescribers are informed. Integrating validated scales into routine clinical practice [36, 37] can objectively document symptom progression to support decisions about antidepressant initiation and discontinuation. Medicine reviews should be scheduled regularly in people with dementia to consider the risks and benefits of all long-term medication.

## Conclusions

In a cohort from London, UK, over a quarter of people recently diagnosed with dementia were prescribed an antidepressant, and around half of these started around the time of the dementia diagnosis. There is a consensus that keeping medication burden as low as possible is preferable in people with dementia, and therefore social support and psychological treatments would be preferred over medication, although this requires the availability of appropriate services. Once antidepressants are started, they are usually continued, so there is an important role for primary and secondary care collaboration at the threshold of dementia to prevent initial prescribing, and a need for universal annual dementia medication reviews to stop any medications doing more harm than good.

## Supplementary Material

aa-26-0287-File003_afag221

## Data Availability

The data used in this study are available at CRIS at South London and Maudsley BRC hub, but restrictions apply for data confidentiality, see https://www.maudsleybrc.nihr.ac.uk/facilities/clinical-record-interactive-search-cris/information-for-researchers/

## References

[1] National Institute for Health and Care Excellence (NICE) . Dementia: Assessment, Management and Support for People Living with Dementia and their Carers. NICE London 2018. https://www.nice.org.uk/guidance/ng97/evidence/full-guideline-pdf-48526 30011160

[2] Cloak N, Schoo C, Al KY. Behavioral and psychological symptoms in dementia. In: *StatPearls [Internet]*, 2024. StatPearls Publishing. https://www.ncbi.nlm.nih.gov/books/NBK551552/31855379

[3] Steck N, Cooper C, Orgeta V. Investigation of possible risk factors for depression in Alzheimer's disease: a systematic review of the evidence. J Affect Disord 2018;236:149–56. 29734098 10.1016/j.jad.2018.04.034

[4] Dafsari FS, Jessen F. Depression—an underrecognized target for prevention of dementia in Alzheimer’s disease. Transl Psychiatry 2020;10:160. 32433512 10.1038/s41398-020-0839-1 PMC7239844

[5] Costello H, Roiser JP, Howard R. Antidepressant medications in dementia: evidence and potential mechanisms of treatment-resistance. Psychol Med 2023;53:654–67. 36621964 10.1017/S003329172200397X PMC9976038

[6] Kim HK, Nunes PV, Oliveira KC et al. Neuropathological relationship between major depression and dementia: a hypothetical model and review. Prog Neuropsychopharmacol Biol Psychiatry 2016;67:51–7. 26780170 10.1016/j.pnpbp.2016.01.008

[7] Mars B, Heron J, Kessler D et al. Influences on antidepressant prescribing trends in the UK: 1995–2011. Soc Psychiatry Psychiatr Epidemiol 2017;52:193–200. 27885400 10.1007/s00127-016-1306-4 PMC5329088

[8] Lunghi C, Cosimo AI, Sofia B et al. Prevalence and determinants of long-term utilization of antidepressant drugs: a retrospective cohort study. Neuropsychiatr Dis Treat 2020;16:1157–70. 32440131 10.2147/NDT.S241780 PMC7213896

[9] Dong M, Liu C, Luo H et al. Efficacy and tolerability of antidepressants monotherapy for behavioral and psychological symptoms of dementia: a meta-analysis of randomized controlled trials. J Psychiatr Res 2025;181:417–24. 39662328 10.1016/j.jpsychires.2024.12.005

[10] Mo M, Abzhandadze T, Hoang MT et al. Antidepressant use and cognitive decline in patients with dementia: a national cohort study. BMC Med 2025;23:82. 39994788 10.1186/s12916-025-03851-3 PMC11854023

[11] Davis KAS, Mueller C, Ashworth M et al. What gets recorded, counts: dementia recording in primary care compared with a specialist database. Age Ageing 2021;50:2206–13. 34417796 10.1093/ageing/afab164 PMC8581382

[12] Perera G, Broadbent M, Callard F et al. Cohort profile of the South London and Maudsley NHS Foundation Trust biomedical research Centre (SLaM BRC) case register: current status and recent enhancement of an electronic mental health record-derived data resource. BMJ Open 2016;6:e008721. 10.1136/bmjopen-2015-008721 PMC4785292 26932138

[13] Ashworth M, Durbaba S, Whitney D et al. Journey to multimorbidity: longitudinal analysis exploring cardiovascular risk factors and sociodemographic determinants in an urban setting. BMJ Open 2019;9:e031649. 10.1136/bmjopen-2019-031649 PMC7008443 31874873

[14] Royal Pharmaceutical Society of Great Britain . *British National Formulary*. National Institute for Health and Care Excellence, 2024. https://bnf.nice.org.uk/

[15] Charlson ME, Pompei P, Ales KL et al. A new method of classifying prognostic comorbidity in longitudinal studies: development and validation. J Chronic Dis 1987;40:373–83. 3558716 10.1016/0021-9681(87)90171-8

[16] Burns A, Beevor A, Lelliott P et al. Health of the nation outcome scales for elderly people (HoNOS 65+). Br J Psychiatry 1999;174:424–7. 10616609 10.1192/bjp.174.5.424

[17] John A, McGregor J, Fone D et al. Case-finding for common mental disorders of anxiety and depression in primary care: an external validation of routinely collected data. BMC Med Inform Decis Mak 2016;16:35. 26979325 10.1186/s12911-016-0274-7 PMC4791907

[18] NIHR Maudsley Biomedical Research Centre (BRC) . Natural Language Processing (NLP) Service. 2024. https://www.maudsleybrc.nihr.ac.uk/facilities/clinical-record-interactive-search-cris/cris-natural-language-processing/

[19] Benjamini Y, Heller R, Yekutieli D. Selective inference in complex research. Philos Trans R Soc A 2009;367:4255–71. 10.1098/rsta.2009.0127 PMC3263782 19805444

[20] Benchimol EI, Smeeth L, Guttmann A et al. The REporting of studies Conducted using Observational Routinely-collected health Data (RECORD) statement. PLoS Med 2015;12:e1001885. 26440803 10.1371/journal.pmed.1001885 PMC4595218

[21] vom Hofe I, Stricker BH, Vernooij MW et al. Antidepressant use in relation to dementia risk, cognitive decline, and brain atrophy. Alzheimers Dement 2024;20:3378–87. 38561253 10.1002/alz.13807 PMC11095425

[22] Taylor-Rowan M, Alharthi AA, Noel-Storr AH et al. Anticholinergic deprescribing interventions for reducing risk of cognitive decline or dementia in older adults with and without prior cognitive impairment. Cochrane Database Syst Rev 2023;12:Cd015405. 38063254 10.1002/14651858.CD015405.pub2 PMC10704558

[23] Porsteinsson AP, Drye LT, Pollock BG et al. Effect of citalopram on agitation in Alzheimer disease: the CitAD randomized clinical trial. JAMA. 2014;311:682–91. 24549548 10.1001/jama.2014.93 PMC4086818

[24] Barnes DE, Yaffe K, Byers AL et al. Midlife vs late-life depressive symptoms and risk of dementia: differential effects for Alzheimer disease and vascular dementia. Arch Gen Psychiatry 2012;69:493–8. 22566581 10.1001/archgenpsychiatry.2011.1481 PMC3704214

[25] Nina B, Jennifer B, Manjunadh P et al. Treatment for depression comorbid with dementia. Evidence based mental. Evidence Based Mental Health 2019;22. 10.1136/ebmental-2019-300113 PMC10231626 31558560

[26] Ismail Z, Agüera-Ortiz L, Brodaty H et al. The mild behavioral impairment checklist (MBI-C): a rating scale for neuropsychiatric symptoms in pre-dementia populations. J Alzheimer's Dis 2017;56:929–38. 28059789 10.3233/JAD-160979 PMC5652315

[27] Shao Z, Richie WD, Bailey RK. Racial and ethnic disparity in major depressive disorder. J Racial Ethn Health Disparities 2016;3:692–705. 27294764 10.1007/s40615-015-0188-6

[28] Jones ME, Petersen I, Walters K et al. Differences in psychotropic drug prescribing between ethnic groups of people with dementia in the United Kingdom. Clin Epidemiol 2020;12:61–71. 32021472 10.2147/CLEP.S222126 PMC6980848

[29] Orgeta V, Tabet N, Nilforooshan R et al. Efficacy of antidepressants for depression in Alzheimer’s disease: systematic review and meta-analysis. J Alzheimer's Dis 2017;58:725–33. 28505970 10.3233/JAD-161247 PMC5467718

[30] Bingley J, Young A, Chong TWH. What is the evidence for using antidepressants to reduce anxiety for people with dementia? Arch Gerontol Geriatr Plus 2025;2:100108.

[31] Lo CWH, Gillett AC, Iveson MH et al. Antidepressant switching as a proxy phenotype for drug nonresponse: investigating clinical, demographic, and genetic characteristics. Biological Psychiatry Global Open Science 2025;5:100502. 40510220 10.1016/j.bpsgos.2025.100502 PMC12162021

[32] Dickinson R, Knapp P, House AO et al. Long-term prescribing of antidepressants in the older population: a qualitative study. Br J Gen Pract 2010;60:e144–55. 20353660 10.3399/bjgp10X483913 PMC2845505

[33] Henssler J, Schmidt Y, Schmidt U et al. Incidence of antidepressant discontinuation symptoms: a systematic review and meta-analysis. Lancet Psychiatry 2024;11:526–35. 38851198 10.1016/S2215-0366(24)00133-0

[34] Lee Eric A, Wong C-A, Barrio L et al. An approach to deprescribe antidepressants for depression in older adults: consensus, multidisciplinary practice recommendations. Perm J 27:1–8. 10.7812/TPP/22.052 PMC10266842 36999271

[35] Kusoro O, Roche M, Del-Pino-Casado R et al. Time to diagnosis in dementia: a systematic review with meta-analysis. Int J Geriatr Psychiatry 2025;40:e70129. 40716451 10.1002/gps.70129 PMC12300619

[36] Kaufer DI, Cummings JL, Ketchel P et al. Validation of the NPI-Q, a brief clinical form of the neuropsychiatric inventory. J Neuropsychiatry Clin Neurosci 2000;12:233–9. 11001602 10.1176/jnp.12.2.233

[37] Park S-H, Cho YS. Predictive validity of the Cornell scale for depression in dementia among older adults with and without dementia: a systematic review and meta-analysis. Psychiatry Res 2022;310:114445. 35190341 10.1016/j.psychres.2022.114445

